# Opioid administration across racial and ethnic groups for patients undergoing liver resection: are there disparities?

**DOI:** 10.1186/s13741-024-00473-w

**Published:** 2024-12-02

**Authors:** Blaine Stannard, Allen Ninh, Victoria Mroz, Yuxia Ouyang, Natalia N. Egorova, Samuel DeMaria, Ryan Wang

**Affiliations:** 1 Department of Anesthesiology, Perioperative & Pain Medicine, Icahn School of Medicine at Mount Sinai, 1 Gustave Levy Place, Klingenstein Clinical Center, 8th Floor, New York, NY 10029 USA; 2 Department of Anesthesiology, Perioperative, and Pain Medicine, Stanford University School of Medicine, Palo Alto, CA USA; 3 Department of Population Health Science and Policy, Icahn School of Medicine at Mount Sinai, New York, NY USA

**Keywords:** Disparities, Acute pain, Pain management, Equity, Hepatobiliary

## Abstract

**Background:**

Racial and ethnic disparities in the treatment of perioperative pain have not been well-studied, despite being observed in a variety of other medical settings. The goal of this investigation was to evaluate the relationship between race and ethnicity and intra- and postoperative opioid administration for patients undergoing open liver resection surgery.

**Methods:**

In this single-center retrospective cohort study, adult patients undergoing open liver resection from January 2012 to May 2019 were identified. Demographic, intraoperative, and postoperative data were extracted from the institutional perioperative data warehouse. The primary outcome was weight-based intraoperative morphine milligram equivalents (MME/kg). Secondary outcome variables included use of neuraxial analgesia and length of stay (LOS). Multivariable regression models were used, which controlled for pertinent factors such as age and duration of surgery.

**Results:**

There were 1294 adult open liver resections included in this study: 532 (41%) patients self-reported as White, 401 (31%) as Asian, 159 (12%) as Black, 97 (7%) as Hispanic, and 105 (8%) as Other. The risk adjusted mean intraoperative MME/kg was not different among racial groups (White: 3.25 [95% CL 3.02–3.49] mg/kg vs. Asian: 3.38 [95% CL 3.10–3.69] mg/kg, *p* = 0.87; Black: 2.95 [95% CL 2.70–3.23] mg/kg, *p* = 0.19; Hispanic: 3.36 [95% CL 3.00–3.77] mg/kg, *p* = 0.97). In the multivariable models for secondary outcomes, length of stay was significantly higher for Black (estimate: 1.17, CL: 1.00 to 1.35, *p* = 0.047) and Hispanic (1.30, CL: 1.05 to 1.65, *p* = 0.018) patients relative to White patients. No racial/ethnic groups were significantly associated with higher or lower odds of receiving regional anesthesia.

**Conclusions:**

For patients undergoing liver resection surgery, no racial and ethnic disparities were observed for weight-based intraoperative MME.

**Supplementary Information:**

The online version contains supplementary material available at 10.1186/s13741-024-00473-w.

## Introduction

Racial and ethnic disparities in the treatment of pain have been observed in a variety of medical settings, spanning from the emergency department to outpatient chronic pain management (Anderson et al. 2009; Green et al. 2003; Rahavard et al. 2017; Pletcher et al. 2008). The literature examining the effect of race and ethnicity on opioid administration in the perioperative setting is relatively sparse, but the available data suggest wide variations in opioid administration that may have racial/ethnic drivers (Ng et al. 1996; Gebre et al. 2023; Sadhasivam et al. 2012). Ng et al. found African American and Hispanic patients received significantly less postoperative total morphine than White patients after open reduction and internal fixation procedures for limb fractures (Ng et al. 1996). In another study among patients undergoing a variety of surgeries under general anesthesia, it was found that African American patients received significantly less intraoperative opioids than other racial/ethnic groups (Gebre et al. 2023). Additionally, in pediatric studies it has been shown that non-White children are less likely to receive non-opioid analgesics during surgery, and consequently may experience greater postoperative pain and require more postoperative analgesic interventions when compared to White children (Jette et al. 2021).

Racial and ethnic disparities in the diagnosis and/or treatment of pain are likely multifactorial and are influenced by patient, provider, and health system factors. Language barriers may prevent patients from adequately reporting their pain and requesting appropriate treatment, and cultural differences in the experience, coping, and communication of pain all play a role (Meints et al. 2019). Racial and ethnic disparities in the type of anesthetic techniques performed such as neuraxial analgesia have also been reported (Glance et al. 2007). There are data demonstrating differences in pain tolerance and sensitivity to similar stimuli across racial and ethnic groups, (Ahn et al. 2017; Rowell et al. 2011) and genetic polymorphisms may contribute to differences in drug metabolism (Liu et al. 2014; Yuan et al. 2015; Zhang et al. 2017). These data may lead some providers to believe certain groups of patients experience more or less pain, with little personalized evidence. Hence, the landscape of providing adequate and appropriate analgesia in the perioperative period remains fraught with difficult questions.

Here we present a retrospective cohort study evaluating the effect of race and ethnicity on intraoperative opioid administration and the use of analgesic adjuncts during open liver resection surgery. This cohort was selected because major hepatic surgery typically necessitates an extensive surgical incision that is typically painful, and the use of opioids, neuraxial analgesia, and/or non-opioid analgesic adjuncts are all common. We hypothesized that there are differences in intraoperative opioid administration between racial and ethnic groups in this population despite controlling for pertinent patient and operative factors.

## Materials and methods

This single-center retrospective cohort study was approved by the Program for the Protection of Human Subjects at our institution with a waiver of written consent: IRB# 19–01166-CR001. Adult patients undergoing open liver resection from January 2012 to May 2019 were identified using CPT codes 47120, 47122, 47125, and 47130. Any type of open liver resection regardless of the number of segments resected, including wedge resections, were included. Donor hepatectomies for living donor liver transplantation were excluded. Demographic, intraoperative, and postoperative data were extracted from the institutional perioperative data warehouse. The data warehouse consolidates information from both our historical anesthesia information management system and our electronic health record (Epic; Epic Systems, Verona, WI, USA).

Race and ethnicity information was self-reported by participants during the admission process, which was subsequently organized into a composite race/ethnicity variable, adhering to adapted standards from the US Census Bureau/Office of Management and Budget. The resulting variable encompassed categories such as White, Black, Hispanic/Latinx, Asian, and Other race/ethnicity. Notably, individuals identifying as Hispanic were classified within the Hispanic race/ethnicity group. Due to a limited representation of patients self-identifying as Native American/Alaskan Native and Indian/South Asian in our dataset, these groups were collectively grouped under the umbrella of Other race/ethnicity. Median income was recorded as the median income in the year 2019 according to patient zip code. Postoperative PCA doses could not be accessed and were excluded from calculation of postoperative morphine milligram equivalents (MME). Whenever feasible, we addressed missing data through a manual examination of charts.

The use of regional anesthesia for postoperative analgesia and the type of technique (i.e., spinal, epidural, or peripheral nerve block) used were agreed upon by the patient, anesthesiologist, and surgeon. As part of our departmental standard enhanced recovery protocol, all suitable candidates were offered an analgesic block. If they agreed, 0.25 mg preservative-free morphine was given intrathecally prior to induction of general anesthesia. If an epidural was preferable according to surgical and patient factors, epidural catheters were placed at a T8–T10 level and were managed intraoperatively at the discretion of the attending anesthesiologist, which commonly involved 3–5 mL boluses of 0.125% bupivacaine as needed during periods of perceived painful stimuli (e.g., at incision, times of tachycardia, and/or hypertension).

The primary outcome measure was intraoperative morphine milligram equivalents per kilogram (MME/kg), which was defined as the total oral morphine milligram equivalents of all opioid-based medications administered intraoperatively divided by patient weight in kilograms. Conversions were calculated using the equianalgesic doses presented by Wen et al. (Wen et al. 2022) Methadone equianalgesia was calculated as 1 mg IV methadone: 1 mg IV morphine, and 1 mg IV morphine: 3 mg PO morphine (Weschules and Bain 2008). Secondary outcome variables included use of regional analgesia and length of stay (LOS).

### Statistical analysis

All variables were summarized using the appropriate descriptive statistics. All continuous variables were manually inspected, distribution was checked, and variables were presented as median [interquartile range] and categorical variables were presented as count (percentage). A statistical power calculation was not performed prior to the study, as the sample size was determined based on the available data in the institutional data warehouse. Patient and case characteristics were assessed across self-reported race/ethnicity using Kruskal–Wallis test for continuous, non-normally distributed variables and χ^2^ tests were used for comparison of categorical variables. Additionally, to account for multiple observations from the same subjects within the study period, we used multivariate generalized estimating equations (GEE) model to assess the risk-adjusted outcomes. The independence working correlation matrix was used and the empirical standard errors of the parameter estimates were reported based on the sandwich estimators. The following covariables were included based on clinical impact on analgesic management: age, sex, weight, preoperative platelet, INR, and creatinine values, year of surgery, indication, duration of surgery, intraoperative estimated blood loss, and use of regional anesthesia (epidural, spinal, or peripheral nerve block). Based on the distribution of outcome variables and modified Park test, we used appropriate GEE models. GEE models with gamma distribution and log link were used to estimate length of stay. GEE models with inversed Gaussian distribution were used to estimate intraoperative MME. GEE models with binomial distribution and log link were used to estimate regional analgesia use. To adjust for multiple comparisons, Dunnett-Hsu adjusted *p* values were provided for contrast estimate of race compared to the reference level White. All statistical analyses were executed using SAS 9.4 software (SAS Institute, Cary, NC, USA). *p* value less than 0.05 was considered statistically significant.

## Results

**Fig. 1:**
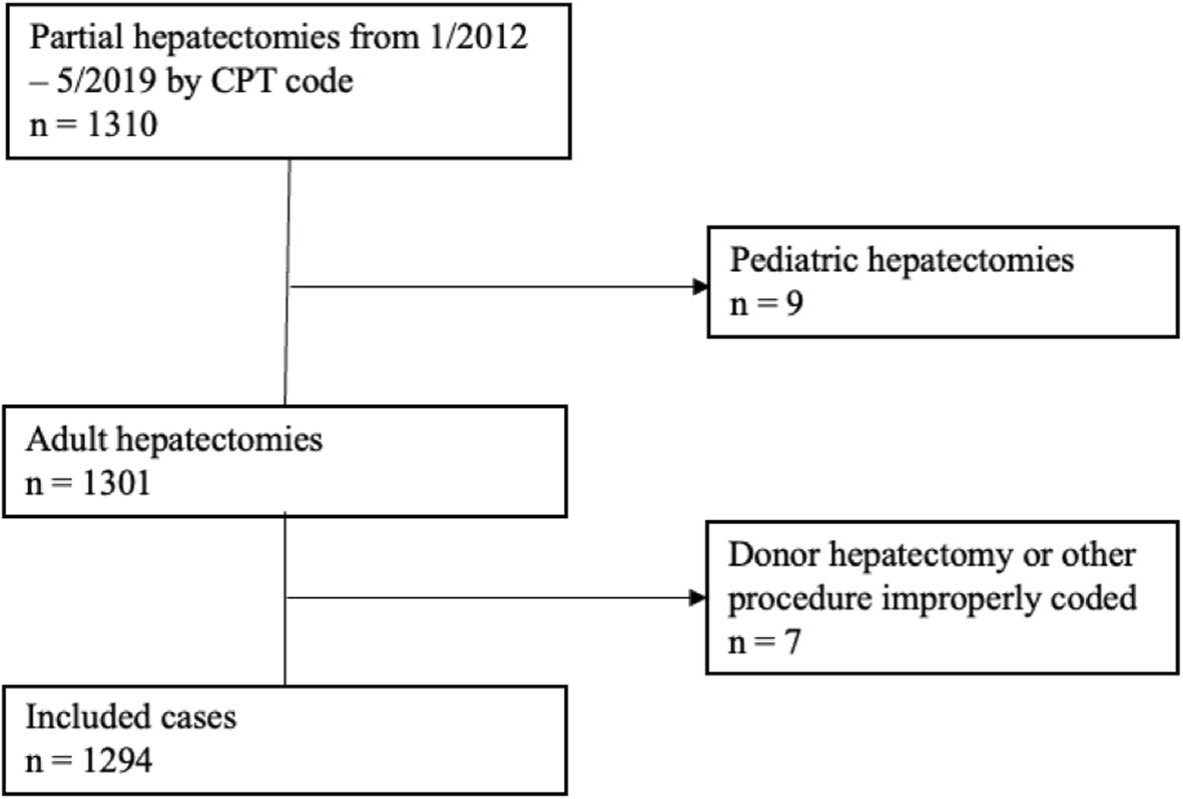
Patient selection diagram

There were 1294 adult open liver resections performed from January 2012 to May 2019, excluding those for living donor liver transplantation (Fig. 1). There were also 30 laparoscopic liver resections during the study period, which were not included in the final cohort of open liver resections. The baseline characteristics of included patients are reported in Table 1. The median patient age was 61 [51–68] years and 556 (43%) were female. There were 532 (41%) patients self-reported as White, 401 (31%) as Asian, 159 (12%) as Black, 97 (7%) as Hispanic, and 105 (8%) as Other. There were significant differences in the median weight (*p* < 0.001), BMI (*p* < 0.001), and median duration of surgery (*p* < 0.001) across racial groups. The most common indications for hepatectomy were hepatocellular carcinoma (643 patients, 50%), non-liver primary malignancy with metastatic disease to the liver (322 patients, 25%), and cholangiocarcinoma (164 patients, 13%).

**Table 1:** Baseline characteristics of patients by race

	Asian (***N*** = 401)	Black (***N*** = 159)	Hispanic (***N*** = 97)	Other (***N*** = 105)	White (***N*** = 532)	***p*** value	All (***N*** = 1294)
Age	60 [51, 68]	61 [51, 66]	60 [51, 69]	59 [47, 67]	63 [54, 70]	**0.002**	61 [51, 68]
Female	123 (31%)	72 (45%)	41 (42%)	43 (41%)	277 (52%)	** < 0.001**	556 (43%)
BMI (kg/m^2^)	23.1 [20.8, 25.1]	27.3 [23.6, 30.0]	26.8 [23.6, 29.1]	25.8 [22.7, 29.0]	26.4 [22.9, 30.1]	** < 0.001**	25.1 [22.3, 28.6]
Height (cm)	165 [160, 170]	170 [163, 178]	165 [158, 170]	168 [160, 174]	168 [160, 175]	** < 0.001**	168 [160, 173]
Weight (kg)	63.0 [55.5, 70.0]	77.0 [68.6, 90.9]	71.0 [62.0, 79.1]	71.8 [63.0, 82.0]	75.0 [63.1, 89.1]	** < 0.001**	70.0 [60.0, 82.9]
Surgery year						0.95	
2012	64 (16.0%)	34 (21.4%)	20 (20.6%)	18 (17.1%)	100 (18.8%)		236 (18.2%)
2013	78 (19.5%)	24 (15.1%)	12 (12.4%)	17 (16.2%)	85 (16.0%)		216 (16.7%)
2014	61 (15.2%)	21 (13.2%)	11 (11.3%)	18 (17.1%)	73 (13.7%)		184 (14.2%)
2015	60 (15.0%)	22 (13.8%)	16 (16.5%)	15 (14.3%)	76 (14.3%)		189 (14.6%)
2016	49 (12.2%)	22 (13.8%)	11 (11.3%)	16 (15.2%)	69 (13.0%)		167 (12.9%)
2017	51 (12.7%)	22 (13.8%)	16 (16.5%)	11 (10.5%)	70 (13.2%)		170 (13.1%)
2018	31 (7.7%)	12 (7.5%)	9 (9.3%)	5 (4.8%)	50 (9.4%)		107 (8.3%)
2019	7 (1.7%)	2 (1.3%)	2 (2.1%)	5 (4.8%)	9 (1.7%)		25 (1.9%)
Diagnosis						** < 0.001**	
Hepatocellular carcinoma	301 (75%)	77 (48%)	32 (33%)	48 (46%)	185 (35%)		643 (50%)
Non-liver primary	30 (8%)	42 (26%)	27 (28%)	27 (26%)	196 (37%)		322 (25%)
Cholangiocarcinoma	34 (8%)	18 (11%)	19 (20%)	12 (11%)	81 (15%)		164 (13%)
Gallbladder carcinoma	6 (2%)	3 (2%)	8 (8%)	2 (2%)	15 (3%)		34 (3%)
NET	3 (1%)	6 (4%)	1 (1%)	0 (0%)	9 (2%)		19 (2%)
Other	27 (7%)	13 (8%)	10 (10%)	16 (15%)	46 (9%)		112 (9%)
Preoperative lab values							
INR	1.0 [0.9, 1.0]	1.0 [1.0, 1.1]	1.0 [1.0, 1.1]	1.0 [0.9, 1.1]	1.0 [0.9, 1.1]	** < 0.001**	1.0 [0.9, 1.1]
Platelets (× 10^9^/L)	193 [151, 247]	220 [165, 275]	223 [181, 296]	209 [167, 262]	217 [167, 277]	** < 0.001**	210 [159, 269]
Creatinine (mg/dL)	0.9 [0.8, 1.0]	1.0 [0.8, 1.1]	0.8 [0.7, 1.0]	0.8 [0.7, 1.0]	0.8 [0.7, 1.0]	** < 0.001**	0.9 [0.7, 1.0]
Surgery duration (min)	175 [127, 232]	205 [146, 257]	212 [157, 281]	192 [153, 235]	210 [151, 290]	** < 0.001**	196 [143, 265]
RBC transfusion required	37 (9%)	17 (11%)	16 (16%)	13 (12%)	71 (13%)	0.20	154 (12%)

Data are as provided number (percentage), median [interquartile range]. *p* values < 0.05 are bolded

*BMI* body mass index, *NET* neuroendocrine tumor, *RBC* red blood cell

Regional anesthesia (including spinal, epidural, and peripheral nerve blocks) was administered in 65% of cases (840 cases), and there was not a significant difference in overall regional anesthesia use across racial groups (*p* = 0.54). The type of neuraxial used did vary across groups, with Asian patients receiving a relatively higher rate of spinal anesthesia and lower rate of epidural anesthesia than the other groups. Peripheral nerve blocks, most commonly transversus abdominis plane (TAP) blocks, were used at similar rates across all groups (Table 2).

**Table 2:** Perioperative analgesic management and outcomes by race

	**Asian (*N* = 401)**	**Black (*N* = 159)**	**Hispanic (*N* = 97)**	**Other (*N* = 105)**	**White (*N* = 532)**	***p* value**	**All (*N* = 1294)**
Regional analgesia used							
Spinal	218 (54%)	68 (43%)	39 (40%)	47 (45%)	224 (42%)	**0.002**	596 (46%)
Epidural	26 (6%)	25 (16%)	16 (16%)	17 (16%)	87 (16%)	** < 0.001**	171 (13%)
Peripheral nerve block	27 (7%)	11 (7%)	6 (6%)	12 (11%)	32 (6%)	0.39	88 (7%)
Regional analgesia used (spinal, epidural, or peripheral)	265 (66.1%)	102 (64.2%)	60 (61.9%)	75 (71.4%)	338 (63.5%)	0.54	840 (64.9%)
Intraoperative MME/kg	3.15 [2.21, 4.29]	2.96 [2.00, 3.85]	3.09 [2.14, 4.80]	3.24 [2.41, 4.29]	3.00 [2.09, 4.37]	0.26	3.05 [2.14, 4.31]
Postoperative PCA used	338 (84%)	122 (77%)	77 (79%)	75 (71%)	424 (80%)	0.07	1036 (80%)
Postoperative LOS (days)	4.68 [3.74, 5.98]	5.09 [3.98, 7.07]	5.07 [3.84, 6.93]	4.96 [3.94, 6.65]	4.88 [3.77, 6.83]	**0.01**	4.91 [3.82, 6.67]

Data are as provided number (percentage), median [interquartile range]. *p* values < 0.05 are bolded

*LOS* length of stay, *MME* milligram morphine equivalents, *PCA* patient-controlled analgesia

As shown in Table 2, unadjusted median intraoperative MME/kg was not significantly different across racial/ethnic groups (Asian patients: 3.15 [2.21–4.29], Black patients: 2.96 [2.00–3.85], Hispanic patients: 3.09 [2.14–4.80], White patients: 3.00 [2.09–4.37], and Other race: 3.24 [2.41–4.29], *p* = 0.26). The rate of postoperative PCA ordered was similar across groups, ranging from 71 to 84%, *p* = 0.07. Median postoperative LOS was slightly higher for Black (5.09 [3.98–7.07] days) and Hispanic (5.07 [3.84–6.93]) patients than White (4.88 [3.77–6.83]) and Asian (4.68 [3.74–5.98]) patients, *p* = 0.01.

As shown in Table 3, the risk adjusted mean intraoperative MME/kg was not different among racial groups (White: 3.25 [95% CL 3.02–3.49] mg/kg vs. Asian: 3.38 [95% CL 3.10–3.69] mg/kg, *p* = 0.87; Black: 2.95 [95% CL 2.70–3.23] mg/kg, *p* = 0.19; Hispanic: 3.36 [95% CL 3.00–3.77] mg/kg, *p* = 0.97). The effect sizes of the covariables included in the generalized estimating equation model for intraoperative MME/kg are shown in Table 4. Covariables significantly associated with MME/kg included age, sex, year of procedure, regional anesthesia use, creatinine, and surgery duration. Of note, regional analgesia was associated with higher intraoperative MME/kg (estimate: 1.47, CL: 1.38–1.57, *p* < 0.001).

**Table 3:** Risk adjusted intraoperative MME

Race	Risk adjusted mean intraoperative MME/kg	Dunnett-Hsu adj *p*
Asian	3.38 (3.10, 3.69)	0.87
Black	2.95 (2.70, 3.23)	0.19
Hispanic	3.36 (3.00, 3.77)	0.97
Other	3.33 (2.99, 3.72)	0.99
White	3.25 (3.02, 3.49)	–

The White patient cohort was used as the reference group

*MME* milligram morphine equivalents. Values are presented as mean (95% confidence level)

**Table 4:** Generalized estimating equation models for intraoperative MME/kg

Predictors	Intraoperative MME/kg		
	Estimates	95% CI	***p*** value
Race			
White	–	–	–
Asian	1.04	0.94–1.15	0.42
Black	0.91	0.82–1.00	0.054
Hispanic	1.03	0.91–1.18	0.60
Other	1.03	0.91–1.16	0.67
Age	1.00	0.99–1.00	**0.004**
Sex (male)	0.90	0.83–0.97	**0.01**
Surgery year			
2012	–	–	–
2013	1.06	0.96–1.18	0.25
2014	1.25	1.12–1.39	** < 0.001**
2015	1.31	1.19–1.43	** < 0.001**
2016	1.13	1.01–1.25	**0.03**
2017	1.16	1.00–1.34	**0.04**
2018	1.16	0.95–1.42	0.15
2019	0.76	0.62–0.93	**0.01**
Diagnosis			
HCC	–	–	–
Cholangiocarcinoma	0.98	0.87–1.12	0.81
Gallbladder carcinoma	0.88	0.73–1.06	0.18
Non-liver primary	0.94	0.86–1.03	0.21
Other	0.80	0.71–0.90	** < 0.001**
Regional analgesia used	1.47	1.38–1.57	** < 0.001**
INR	0.89	0.68–1.14	0.35
Platelets (× 10^9^/L)	0.999	0.996–1.0002	0.48
Creatinine (mg/dL)	0.96	0.94–0.97	** < 0.001**
Surgery duration (min)	1.0018	1.0015–1.0012	** < 0.001**

The White patient cohort, surgery in the year 2012, and a diagnosis of cholangiocarcinoma were used as reference groups. PCA use and postoperative analgesics were not included in the model for intraoperative MME/kg. *p* values < 0.05 are bolded

*INR* international normalized ratio, *HCC* hepatocellular carcinoma, *PCA* patient-controlled analgesia

In the multivariable model shown in Supplemental Table 1, length of stay was significantly higher for Black (estimate: 1.17, CL: 1.00 to 1.35, *p* = 0.047) and Hispanic (1.30, CL: 1.05 to 1.65, *p* = 0.018) patients relative to White patients. No significant difference was observed for Asian patients.

Multivariable logistic regression models examining factors associated with the use of neuraxial anesthesia are shown in Supplemental Table 2. No racial/ethnic groups were significantly associated with higher or lower odds of receiving regional anesthesia after Dunnett-Hsu comparison adjustment.

## Discussion

Disparities in pain management have been identified across racial and ethnic groups but have not been well-studied in the perioperative period. In this study of 1294 patients undergoing open liver resection surgery at a single large academic center, no significant differences in intraoperative MME/kg were observed across racial and ethnic groups. These findings persisted despite controlling for a variety of patient, comorbidity, and operative factors.

Prior investigations have identified differences in postoperative opioid administration across racial and ethnic groups (Ng et al. 1996; Gebre et al. 2023; Konstantatos et al. 2012; Poehlmann et al. 2022). Konstantatos et al. found that in a matched analysis of 128 patients receiving morphine patient-controlled analgesia after major abdominal surgery, Asian patients in Hong Kong utilized less postoperative opioids and had higher pain scores compared to White patients in comparator group from Australia. (Konstantatos et al. 2012) Patient survey responses indicated Asian patients preferred others to manage their pain and expected more severe pain postoperatively. Cultural biases and systemic factors likely contribute to disparities in postoperative pain management, especially when patients must advocate for themselves, such as in requesting additional doses of analgesics. It is notable that none of these studies accounted for intraoperative analgesic management, which both affects postoperative analgesia and can contribute to differences in care (Santa Cruz Mercado et al. 2023).

In a study by Gebre et al. examining a variety of surgeries performed under general anesthesia, African American patients received significantly less intraoperative opioids than other racial/ethnic groups (Gebre et al. 2023). However, the mean intraoperative MME in their study was 50 MME or less across racial/ethnic groups, compared to a median MME of over 200 in our study. It is possible that for surgeries perceived to be more painful, higher intraoperative MME are administered regardless of race which reduces the racial differences. In contrast, a large retrospective study of 21,229 pediatric patients performed by Jette et al. found no difference in intraoperative MME/kg administered across racial groups. They did find that Asian and Hispanic patients were significantly less likely to receive intraoperative non-opioid analgesics compared to White patients. Future studies investigating perioperative MME differences across racial groups should consider intraoperative and weight-adjusted outcomes as well as focus on specific procedure types. There may be certain surgeries or pathologies that are higher risk for variation in analgesic management, and this would also allow for more targeted interventions to reduce disparities.

There are a variety of factors that can influence perioperative opioid administration. Intraoperative analgesic management in anaesthetized patients is often guided by surrogates for pain (e.g., hemodynamic measurements) as well as pharmacokinetic and pharmacodynamic principals, including weight-based dosing. Total intravenous opioid administration can also be affected by the use of neuraxial anesthesia. Disparities in rates of epidural anesthesia have been reported across racial groups, such as in obstetric populations (Glance et al. 2007). We thus explored the potential difference in rates of neuraxial anesthetic utilization between racial/ethnic groups. Although Asian patients had higher unadjusted rates of spinal analgesia and lower rates of epidural catheters than other groups, the composite rate of regional anesthesia was not significantly difference across racial groups. Additionally, in our multivariable logistic regression model no racial or ethnic group was independently associated with higher or lower odds of receiving regional anesthesia. Interestingly, the use of regional anesthesia was associated with significantly higher intraoperative MME/kg in the multivariable model. It is possible that patients that were planned to undergo more extensive surgeries (and thus require more intraoperative opioids) were more likely to receive a regional technique. Additionally, the dose of spinal morphine was 0.25 mg regardless of patient weight and was included in the total MME/kg calculation, which may have affected the MME/kg results.

Developing protocols for pain management could help standardize treatment. Enhanced Recovery After Surgery (ERAS) protocols have been implemented across many hospitals, and there is evidence that they help address disparities in pain management across racial/ethnic groups after surgeries such as cesarean deliveries and congenital cardiac surgery (Felder et al. 2022; Buchanan et al. 2022). In our practice, it is expected among anesthesia and surgical teams that neuraxial analgesia is offered to all appropriate patients as part of our departmental ERAS protocol. Interpreter services are readily available in preoperative holding at our institution, which facilitates discussion of risks and benefits across a diverse patient population. Since no truly objective metrics exist to guide intraoperative opioid administration, standardizing interventions like neuraxial anesthesia represents an important avenue to mitigating disparities.

This investigation is limited in several capacities. This study was performed retrospectively which introduces a risk of selection bias. Additionally, certain pertinent variables were not available (e.g., longitudinal long-term postoperative pain scores), with confounders that are not easily identifiable. Although we were able to identify whether patients received patient-controlled analgesia (PCA) postoperatively, the total PCA opioid doses were not recorded in our electronic medical record or data warehouse in a way that allowed for reliable querying. This limited our ability to assess postoperative MME/kg. The rate of PCAs ordered, however, did not vary significantly across racial/ethnic groups. Future studies could be performed prospectively to explore the relationship between racial/ethnic groups, perioperative pain management, and the development of chronic pain or opioid misuse.

Overall, we found that intraoperative MME/kg was not significantly different across racial and ethnic groups during open liver resection surgery. It is important to recognize disparities in the diagnosis and management of acute pain to improve patient experience and outcomes. The consistency across patient groups in our institution is reassuring, and further study is warranted to identify practices and protocols that facilitate equitable care during major surgery.

## Supplementary Information

Supplementary Material 1.

## Data Availability

De-identified data can be made available upon reasonable request to the authors.

## Abbreviations

GEE: Generalized estimating equations
LOS: Length of stay
MME: Morphine milligram equivalents
PCA: Patient-controlled analgesia
TAP: Transversus abdominis plane
