# Supplementary material for: Opioid administration across racial and ethnic groups for patients undergoing liver resection: are there disparities?
Source: Perioper Med (Lond). 2024 Dec 2;13:114. doi: 10.1186/s13741-024-00473-w (PMC11610172; doi:10.1186/s13741-024-00473-w)
Supplement: Supplementary file 1 — Supplementary Material 1. [file 13741_2024_473_MOESM1_ESM.docx]

**Supplemental Items**

Supplemental Table 1. Generalized estimating equation model for postoperative length of stay.

|  | **Postoperative length of stay** | | |
| --- | --- | --- | --- |
| **Predictors** | **Estimates** | **95% CI** | **p-value** |
| **Race** | | | |
| White | - | - | - |
| Asian | 1.17 | 1.00 – 1.38 | 0.06 |
| Black | 1.22 | 1.06 – 1.41 | **0.01** |
| Hispanic | 1.32 | 1.07 – 1.63 | **0.01** |
| Other | 1.69 | 0.83 – 3.44 | 0.15 |
| **Age** | 1.01 | 1.00 – 1.01 | **0.01** |
| **Sex (male)** | 0.97 | 0.86 – 1.09 | 0.65 |
| **BMI (kg/m^2^)** | 1.00 | 1.00 – 1.00 | 0.70 |
| **Surgery Year** | | | |
| 2012 | - | - | - |
| 2013 | 0.87 | 0.66 – 1.15 | 0.34 |
| 2014 | 0.86 | 0.65 – 1.12 | 0.25 |
| 2015 | 0.90 | 0.69 – 1.16 | 0.41 |
| 2016 | 0.78 | 0.61 – 1.00 | 0.05 |
| 2017 | 0.83 | 0.50 – 1.39 | 0.48 |
| 2018 | 0.67 | 0.51 – 0.87 | **0.003** |
| 2019 | 0.63 | 0.41 – 0.96 | **0.03** |
| **Diagnosis** | | | |
| HCC | - | - | - |
| Cholangiocarcinoma | 1.02 | 0.86 – 1.20 | 0.84 |
| Gallbladder carcinoma | 0.88 | 0.68 – 1.12 | 0.29 |
| Non-liver primary | 0.99 | 0.86 – 1.14 | 0.92 |
| Other | 0.79 | 0.65 – 0.95 | **0.01** |
| **Regional analgesia used** | 1.25 | 1.13 – 1.40 | **<0.001** |
| **Preoperative Lab Values** | | | |
| INR | 2.94 | 1.62 – 5.31 | **<0.001** |
| Platelets (x10^9^/L) | 1.00 | 1.00 – 1.00 | 0.64 |
| Creatinine (mg/dL) | 1.04 | 0.98 – 1.11 | 0.20 |
| **Surgery duration (min)** | 1.00 | 1.00 – 1.00 | **<0.001** |
| **24-hour postoperative Ketorolac (mg)** | 1.00 | 1.00 – 1.00 | 0.56 |
| **24-hour postoperative Acetaminophen (mg)** | 1.00 | 1.00 – 1.00 | 0.91 |

The White patient cohort, surgery in the year 2012, and a diagnosis of hepatocellular carcinoma were used as reference groups. P-values <0.05 are bolded. BMI, body mass index. INR, international normalized ratio. HCC, hepatocellular carcinoma. PCA, patient-controlled analgesia.

Supplemental Table 2. Logistic regression model for use of regional analgesia.

|  | **Regional analgesia used** | | |
| --- | --- | --- | --- |
| **Predictors** | **Odds ratios** | **95% CI** | **p-value** |
| **Race** |  |  |  |
| White | - | - | - |
| Asian | 1.13 | 0.80 – 1.58 | 0.50 |
| Black | 1.01 | 0.66 – 1.55 | 0.97 |
| Hispanic | 0.98 | 0.58 – 1.66 | 0.95 |
| Other | 1.66 | 1.01 – 2.72 | **0.04** |
| **Age** | 0.99 | 0.98 – 1.00 | 0.10 |
| **Sex (male)** | 1.04 | 0.78 – 1.39 | 0.78 |
| **BMI (kg/m^2^)** | 1.00 | 0.99 – 1.02 | 0.44 |
| **Surgery Year** |  |  |  |
| 2012 | - | - | - |
| 2013 | 0.72 | 0.48 – 1.09 | 0.12 |
| 2014 | 1.02 | 0.64 – 1.61 | 0.95 |
| 2015 | 0.80 | 0.53 – 1.21 | 0.29 |
| 2016 | 0.79 | 0.51 – 1.23 | 0.30 |
| 2017 | 1.35 | 0.83 – 2.19 | 0.22 |
| 2018 | 2.82 | 1.60 – 4.96 | **<0.001** |
| 2019 | 3.09 | 1.07 – 8.88 | **0.04** |
| **Diagnosis** |  |  |  |
| HCC | - | - | - |
| Cholangiocarcinoma | 0.72 | 0.46 – 1.12 | 0.14 |
| Gallbladder carcinoma | 0.68 | 0.36 – 1.32 | 0.26 |
| Non-liver primary | 0.85 | 0.60 – 1.21 | 0.37 |
| Other | 0.67 | 0.41 – 1.08 | 0.10 |
| **Preoperative lab values** |  |  |  |
| INR | 0.15 | 0.05 – 0.47 | **0.001** |
| Platelets (x10^9^/L) | 1.00 | 1.00 – 1.00 | 0.78 |
| Creatinine (mg/dL) | 0.97 | 0.79 – 1.19 | 0.76 |
| **Surgery duration (min)** | 1.00 | 1.00 – 1.004 | **0.004** |

The White patient cohort, surgery in the year 2012, and a diagnosis of hepatocellular carcinoma were used as reference groups. P-values <0.05 are bolded. INR, international normalized ratio. HCC, hepatocellular carcinoma. PCA, patient-controlled analgesia.
